# Synthesis and structure of 3-(14*H*-dibenzo[*a*,*j*]xanthen-14-yl)phenyl nicotinate

**DOI:** 10.1107/S2056989026003415

**Published:** 2026-04-10

**Authors:** Ekaterina A. Akishina, Artyom A. Khadarovich, Mikhail S. Grigoriev, Victoria I. Salakhova, Tuncer Hökelek, Khudayar I. Hasanov, Alebel N. Belay

**Affiliations:** a Institute of Physical Organic Chemistry, National Academy of Sciences of Belarus, Minsk, 220072, Belarus; b Frumkin Institute of Physical Chemistry and Electrochemistry, Russian Academy of Sciences, Leninsky prosp. 31, bld. 4, Moscow 119071, Russian Federation; c RUDN University, 6 Miklukho-Maklaya St., Moscow 117198, Russian Federation; d Hacettepe University, Department of Physics, 06800 Beytepe-Ankara, Türkiye; e Azerbaijan Medical University, Scientific Research Centre (SRC), A. Kasumzade St. 14, AZ 1022, Baku, Azerbaijan; f Department of Chemistry, Bahir Dar University, PO Box 79, Bahir Dar, Ethiopia; University of Aberdeen, United Kingdom

**Keywords:** xanthenes, crystal structure, non-covalent inter­actions

## Abstract

In the title compound, C_33_H_21_NO_3_, the dihedral angle between the naphthalene units is 10.85 (4)° and the pyran ring adopts a shallow boat conformation. In the crystal, C—H⋯N and C—H⋯O hydrogen bonds link the mol­ecules, enclosing *R*^2^_2_(16) ring motifs. Hirshfeld surface analysis revealed that the most important contributions for the crystal packing are from H⋯H, H⋯C/C⋯H, H⋯O/O⋯H and C⋯C inter­actions, at 45.8%, 27.3%, 11.2% and 9.6%, respectively.

## Abstract 2

In the title compound, the dihedral angle between the naphthalene units is 10.85 (4)° and the pyran ring adopts a shallow boat conformation. In the crystal, C—H⋯N and C—H⋯O hydrogen bonds link the mol­ecules, enclosing *R*^2^_2_(16) ring motifs.

## 1. Chemical context

Dibenzo[*a*,*j*]xanthenes are heterocyclic aromatic compounds consisting of a central pyran ring fused to two naphthalene units. Owing to their extended π-conjugation and nearly planar structures, these compounds have found applications as photosensitive materials (Brøndsted & Stains 2024 ▸; Rawat *et al.*, 2025 ▸) and DNA inter­calators (Tacar *et al.*, 2013 ▸). In addition, several derivatives exhibit significant biological activity, including anti­bacterial (Amininasab *et al.*, 2020 ▸) and anti­viral properties (Reddi Mohan Naidu *et al.*, 2012 ▸), and have shown potential for use in cancer photodynamic therapy (Smolobochkin *et al.*, 2024 ▸; Wang *et al.*, 2020 ▸; Karaman *et al.*, 2023 ▸).

The condensation of β-naphthol with aldehydes represents one of the most convenient and widely used approaches for the synthesis of these compounds, with catalyst selection being a key factor for reaction efficiency. Over the past several years, we have investigated the application of the sulfonic cation-exchange resin FIBAN K-1 as a catalyst for the efficient synthesis of xanthene derivatives (Akishina *et al.*, 2025 ▸; Akishina *et al.*, 2023 ▸).

Examining the spatial arrangement of dibenzoxanthenes can offer insights into their electronic conjugation and mol­ecular planarity, which are crucial factors affecting fluorescence efficiency, quantum yield and emission wavelength (Ji *et al.*, 2024 ▸). In addition, to evaluate the biological potential of a mol­ecule using the mol­ecular docking method detailed information about the structures of promising mol­ecules is essential (Akishina *et al.*, 2026 ▸).

As part of our ongoing studies in this area, we now describe the synthesis and crystal structure, together with the Hirshfeld surface analysis, of the title compound, C_33_H_21_NO_3_ (**1**).
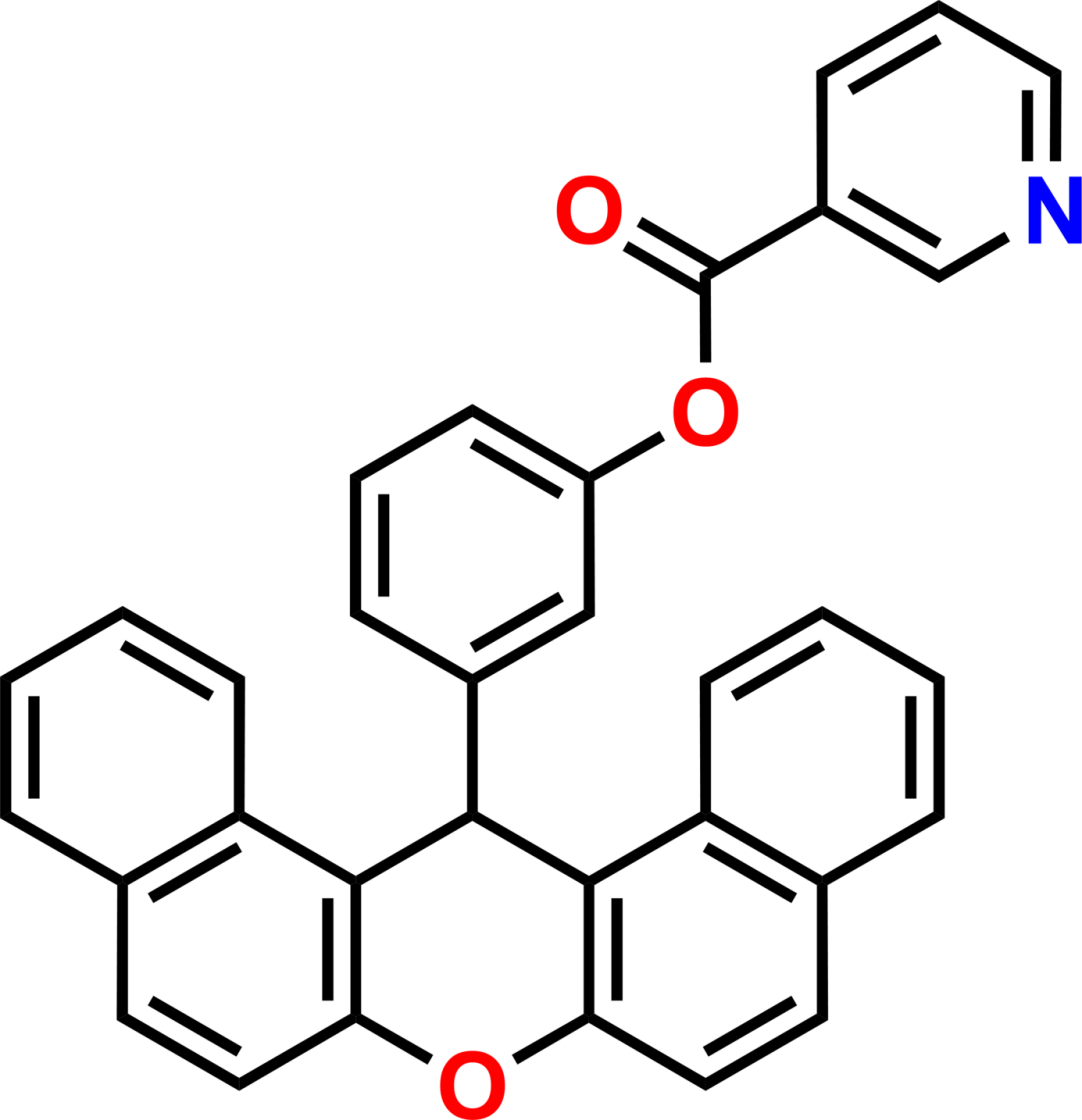


## 2. Structural commentary

**Figure 1:**
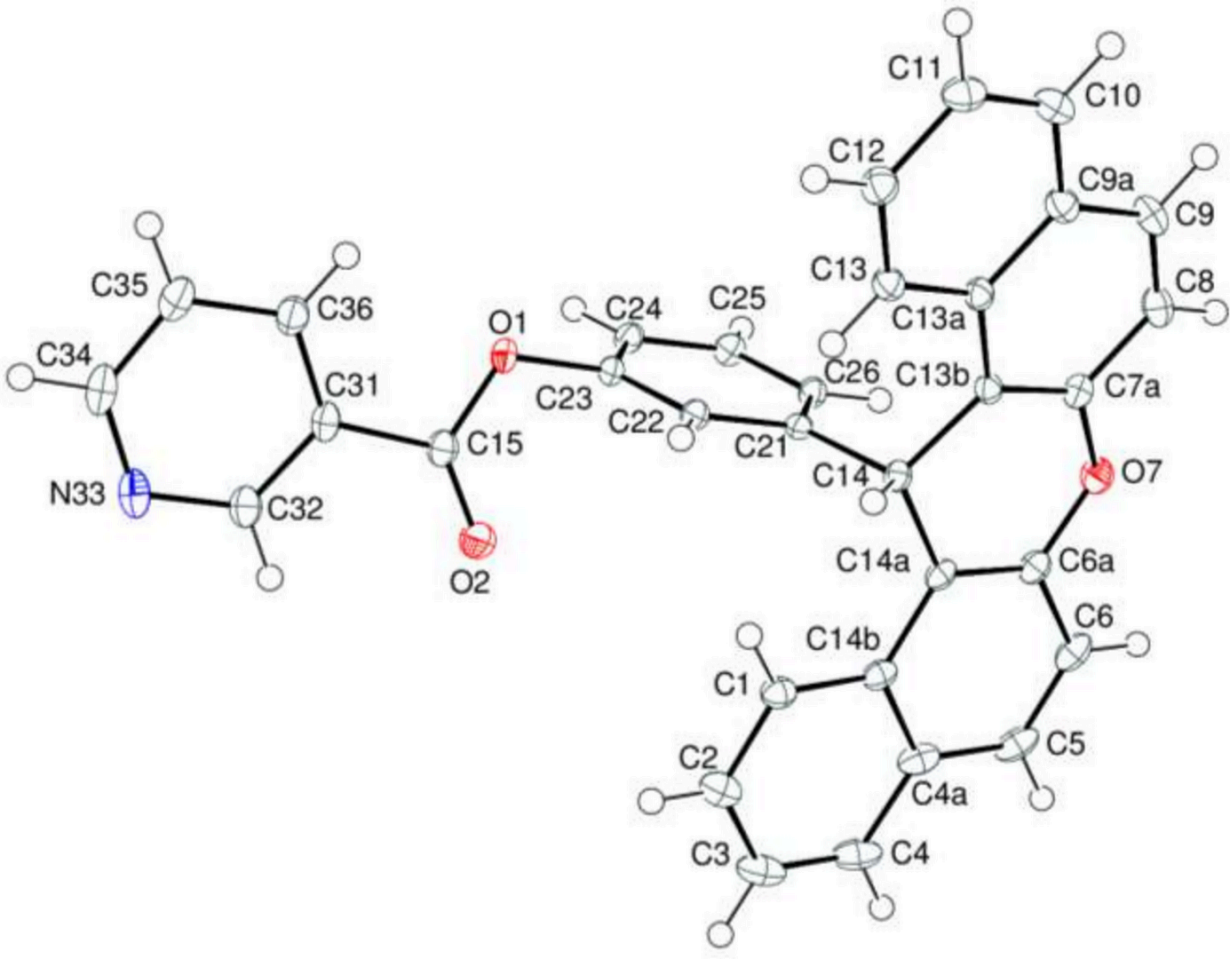
The mol­ecular structure of (**1**) showing 50% displacement ellipsoids.

The asymmetric unit of (**1**) consists of one mol­ecule in space group *P*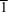. The mol­ecule is constructed from 14*H*-dibenzo[*a*,*j*]xanthene and phenyl nicotinate moieties connected *via* the C14—C21 bond (Fig. 1 ▸). In the first of these, the benzene *A* (C1–C4/C4*A*/C14*B*), *B* (C4*A*/C5/C6/C6*A*/C14*A*/C14*B*), *D* (C7*A*/C8/C9/C9*A*/C13*A*/C13*B*) and *E* (C9*A*/C10–C13/C13*A*) rings are oriented at dihedral angles of *A*/*B* = 1.36 (4)° and *D*/*E* = 1.53 (4)°, indicating a slight puckering of the naphthyl units. The dihedral angle between the naphthyl units is 10.85 (4)°. The non-planar pyran *C* (O7/C6*A*/C7*A*/C13*B*/C14/C14*A*) ring is in a shallow boat conformation with Cremer–Pople puckering parameters of *Q*_T_ = 0.1973 (14) Å, θ = 101.97 (43)° and φ = 10.3 (4)°. Alternately, we may state that atoms O7 and C14 are displaced from the mean plane of atoms C6*A*/C7*A*/C13*B*/C14*A* (r.m.s. deviation = 0.034 Å) by −0.114 (1) and −0.218 (1) Å, respectively. On the other hand, the phenyl *F* (C21–C26) and pyridine *G* (C31–C36/N33) rings are almost perpendicularly oriented at a dihedral angle of *F*/*G* = 83.39 (5)°. The ester O1—C15—O2 [123.75 (12)°] bond angle is slightly increased with respect to that present in the free acid [122.2°] (Sim *et al.*, 1955 ▸).

## 3. Supra­molecular features

**Figure 2:**
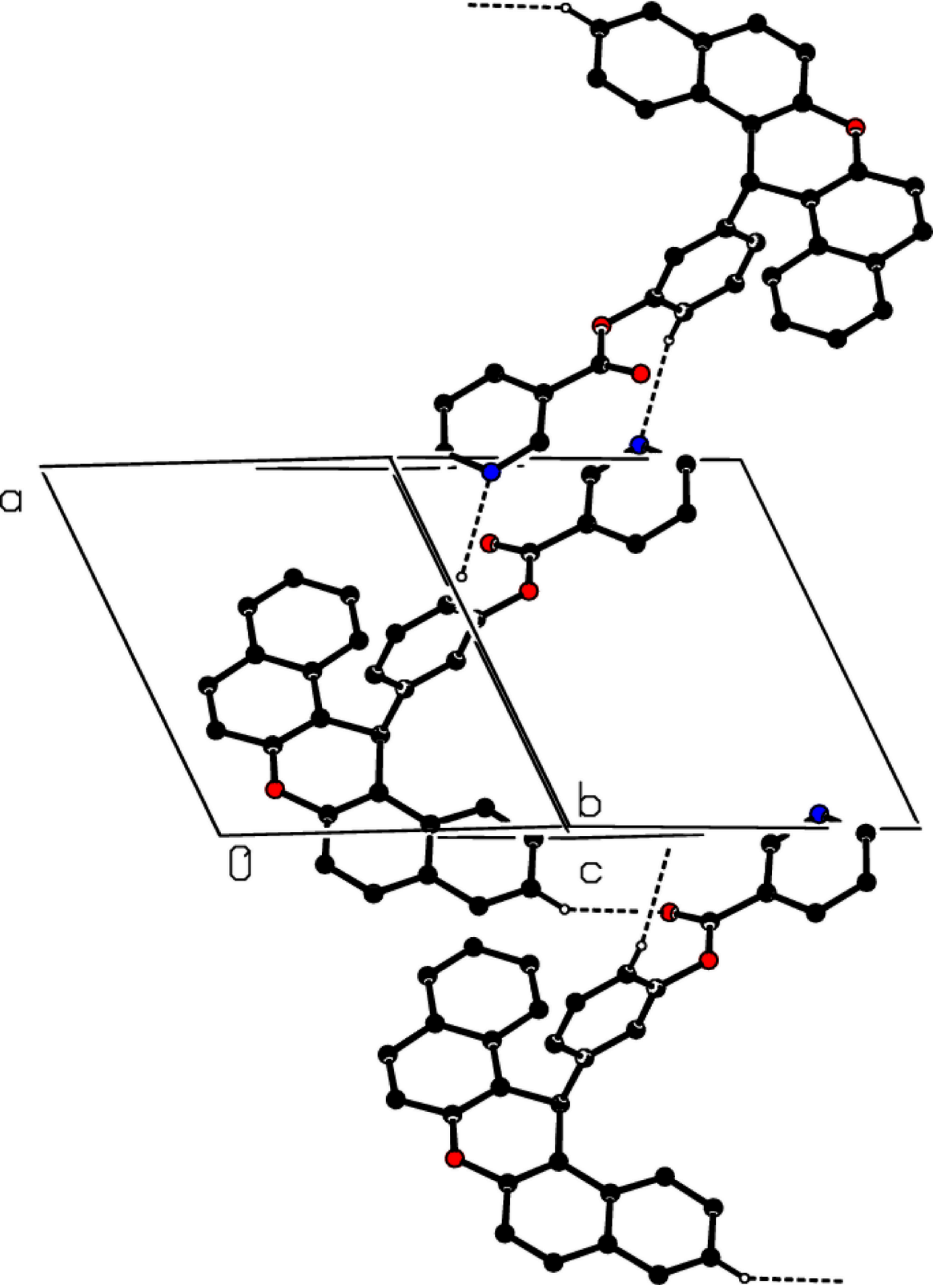
The partial packing diagram of (**1**) with C—H⋯N and C—H⋯O hydrogen bonds shown as dashed lines. H atoms not involved in these inter­actions have been omitted for clarity.

In the crystal, C—H⋯N and C—H⋯O hydrogen bonds (Table 1 ▸) link the mol­ecules, enclosing 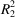
(16) ring motifs (Etter *et al.*, 1990 ▸) (Fig. 2 ▸). In addition, C—H⋯π inter­actions (Table 1 ▸) and weak π–π stacking inter­actions between the *B* and *G*, *A* and *E*, *D* and *E* and between the *E* rings [with centroid-to-centroid distances and α values of 3.7087 (7) Å and 11.53°, 3.8153 (9) Å and 10.29 °, 4.7923 (9) Å and 1.49° and 4.3282 (9) Å and 0.00°, respectively] may help to consolidate the three-dimensional architecture.

## 4. Hirshfeld surface analysis

**Figure 3:**
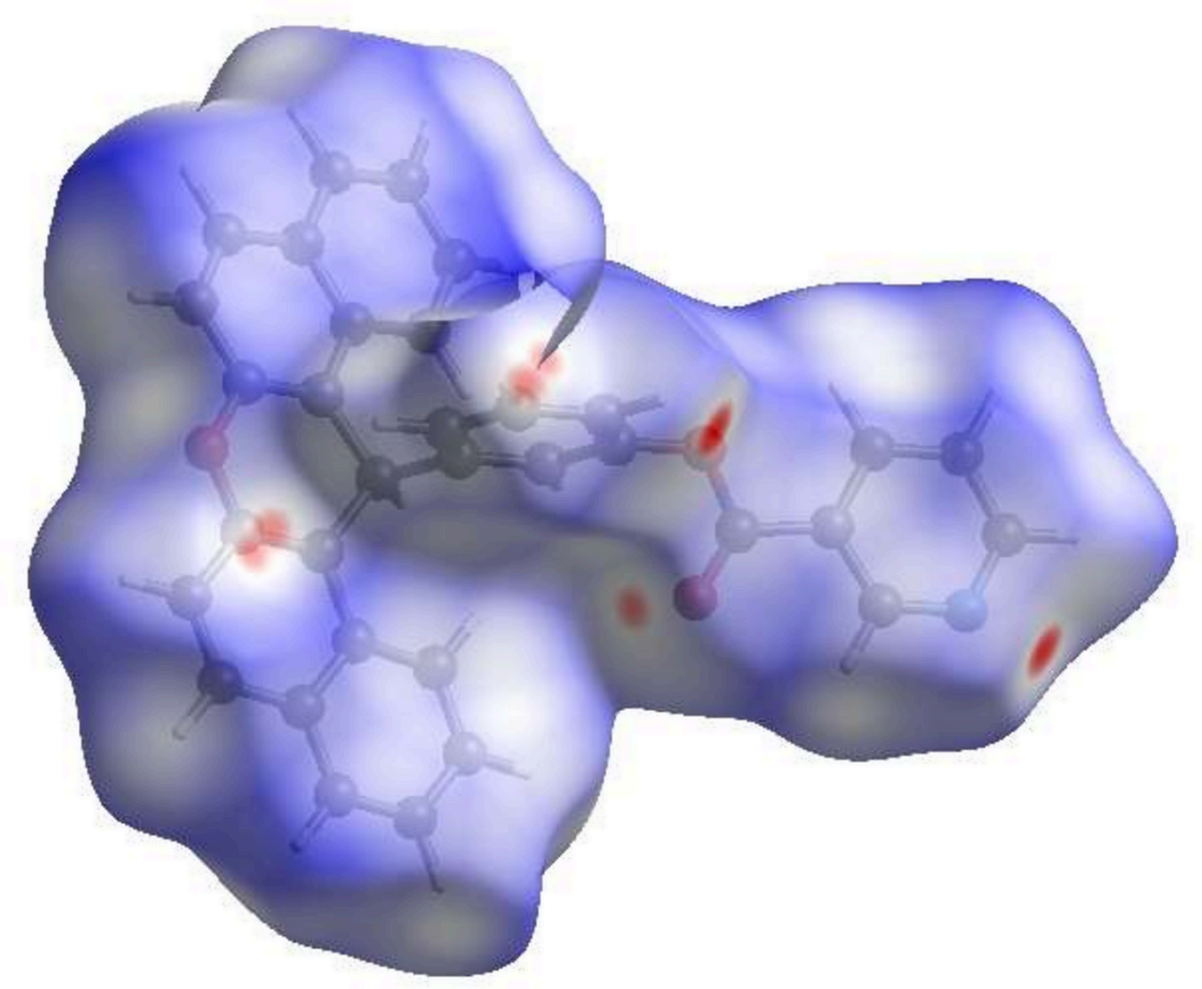
View of the three-dimensional Hirshfeld surface of (**1**) plotted over *d*_norm_ in the range from −0.15 to 1.46 a.u.

**Figure 4:**
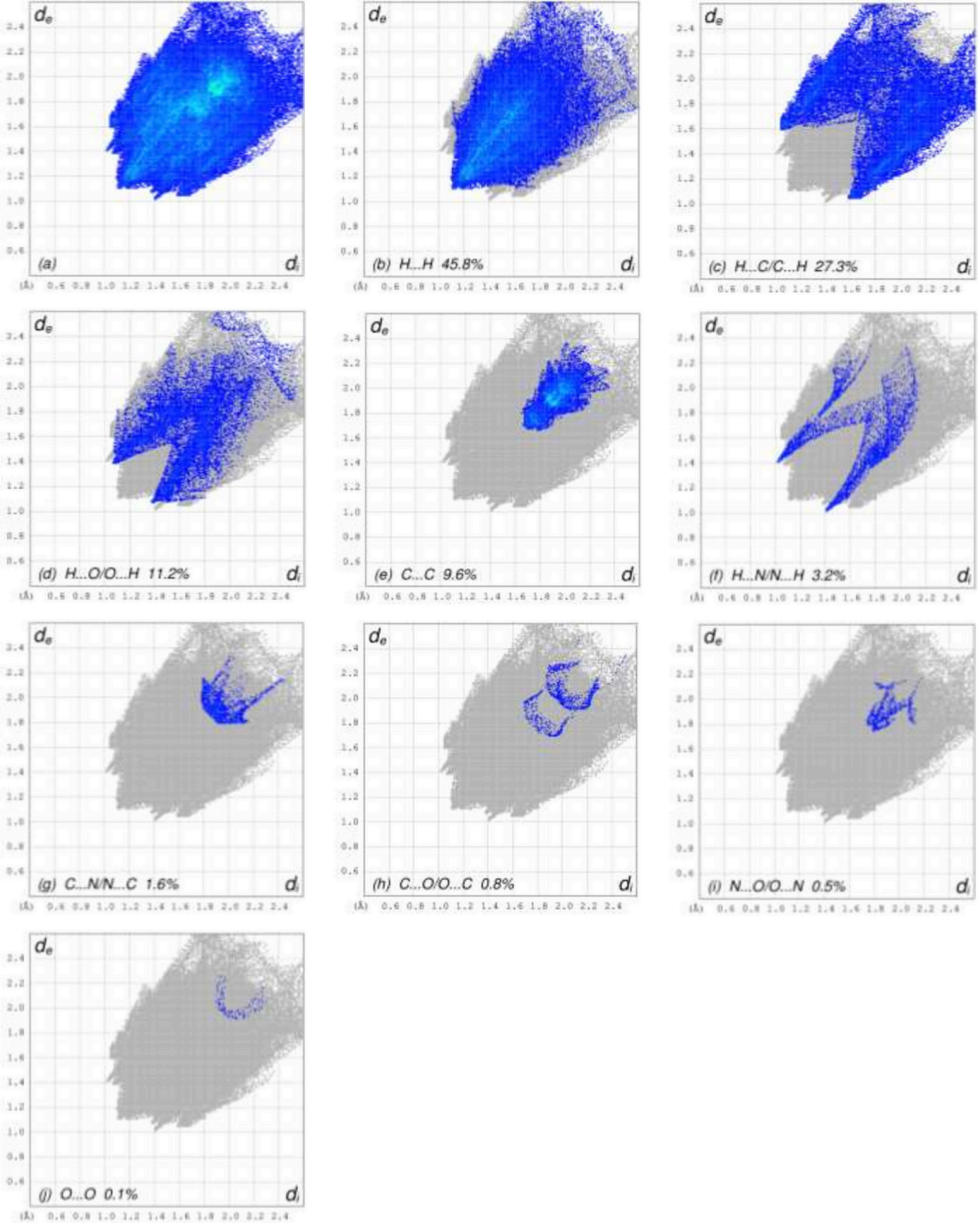
Two-dimensional fingerprint plots for (**1**), showing (a) all inter­actions, and (b*)*–(*j*), delineated into the various contact types. The *d*_i_ and *d*_e_ values are the closest inter­nal and external distances (in Å) from the given points on the Hirshfeld surface.

For visualizing the inter­molecular inter­actions in the crystal of (**1**), Hirshfeld surface (HS) analysis was carried out by using *Crystal Explorer* 17.5 (Spackman *et al.*, 2021 ▸). In the HS plotted over *d*_norm_ (Fig. 3 ▸), the red spots indicate their roles as the respective donors and/or acceptors in hydrogen bonding, as discussed above. The overall two-dimensional fingerprint plot is shown in Fig. 6*a* and those delineated into various contact types are illustrated in Fig. 6*b*–*j*. According to the fingerprint plots, H⋯H, H⋯C/C⋯H, H⋯O/O⋯H and C⋯C contacts make the most significant contributions to the HS, at 45.8%, 27.3%, 11.2% and 9.6%, respectively (Fig. 4 ▸).

## 5. Synthesis and crystallization

**Figure 5:**
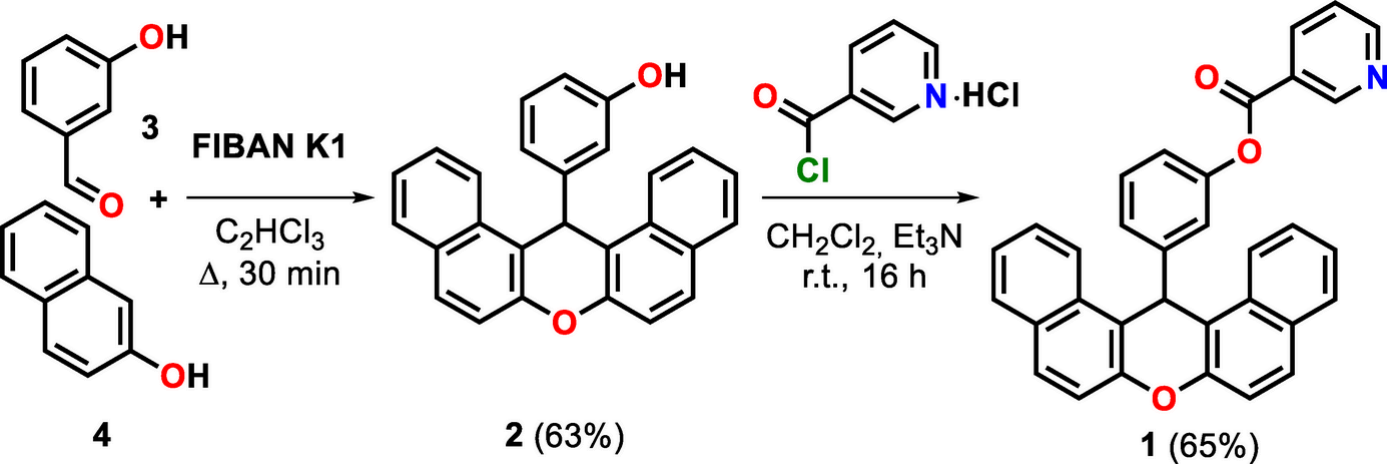
The reaction scheme for obtaining compound (**1**). FIBAN K1 is a fibrous sulfonic cation-exchange resin containing strongly acidic sulfonic acid (–SO_3_H) functional groups immobilized on a polymeric matrix.

Compound **1** was obtained according to the method (Fig. 5 ▸) described by us earlier (Akishina *et al.*, 2025 ▸). A mixture of 3-hy­droxy­benzaldehyde (**3**) (0.30 g, 2.5 mmol) and 2-naphthol (**4**) (0.72 g, 5.0 mmol) in the presence of 1.8 g of FIBAN K-1 was boiled in tri­chloro­ethyl­ene (90 ml) with a Dean–Stark trap for 30 min. The catalyst was filtered off using a sintered glass filter, washed with tri­chloro­ethyl­ene (50 ml), and the solvent was completely removed under reduced pressure. The residue was washed with 40 ml of water–ethanol mixture (1:1), the product was filtered off and dried in vacuum over P_2_O_5_. Nicotinic acid chloride hydro­chloride (0.45 g, 2.5 mmol) was added with stirring to the xanthenyl-substituted phenol (**2**) (0.86 g, 2.3 mmol) and tri­ethyl­amine (0.51 g, 5 mmol) in di­chloro­methane (50 ml). The reaction mixture was stirred for 1 h and left for 15 h at room temperature, washed with water and NaHCO_3_ solution. The organic layer was separated, dried over Na_2_SO_4_ and filtered. The solvent was evaporated, the product was isolated by low-temperature recrystallization from a mixture of ethyl acetate and hexane to give **1** as yellow solid (0.50 g, 65%). m.p. 470–471 K. Yellow crystals of (**1**) suitable for single-crystal X-ray diffraction study were obtained from ethyl acetate solution by slow evaporation at room temperature.

## 6. Refinement

Crystal data, data collection and structure refinement details are summarized in Table 2 ▸. The C-bound hydrogen-atom positions were calculated geometrically at distances of 1.00 (for methine CH) and 0.95 (for aromatic CH) and refined using a riding model by applying the constraint *U*_iso_(H) = 1.2*U*_eq_(C).

## Supplementary Material

Crystal structure: contains datablock(s) I, global. DOI: 10.1107/S2056989026003415/hb8205sup1.cif

Structure factors: contains datablock(s) I. DOI: 10.1107/S2056989026003415/hb8205Isup3.hkl

Spectroscopic data (IR and NMR). DOI: 10.1107/S2056989026003415/hb8205sup4.docx

Supporting information file. DOI: 10.1107/S2056989026003415/hb8205Isup4.cml

CCDC reference: 2543051

Additional supporting information:  crystallographic information; 3D view; checkCIF report

## Figures and Tables

**Table 1:** Hydrogen-bond geometry (Å, °) *Cg*4 and *C*g7 are the centroids of the C4*A*/C5/C6/C6*A*/C14*A*/C14b*B* and C21–C26 rings, respectively.

*D*—H⋯*A*	*D*—H	H⋯*A*	*D*⋯*A*	*D*—H⋯*A*
C24—H24⋯N33^i^	0.95	2.53	3.3298 (18)	142
C11—H11⋯O2^ii^	0.95	2.56	3.329 (2)	138
C11—H11⋯*Cg*7^iii^	0.95	3.05	3.8352 (19)	142
C25—H25⋯*Cg*4^iv^	0.95	3.01	3.9301 (15)	162

Symmetry codes: (i) 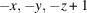; (ii) 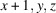; (iii) 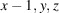; (iv) 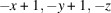.

**Table 2:** Experimental details

Crystal data	
Chemical formula	C_33_H_21_NO_3_
*M*_r_	479.51
Crystal system, space group	Triclinic, *P* 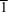
Temperature (K)	100
*a*, *b*, *c* (Å)	9.6572 (6), 11.0020 (7), 12.2442 (8)
α, β, γ (°)	90.489 (2), 100.530 (2), 113.735 (2)
*V* (Å^3^)	1165.95 (13)
*Z*	2
Radiation type	Mo *K*α
μ (mm^−1^)	0.09
Crystal size (mm)	0.36 × 0.32 × 0.20

Data collection	
Diffractometer	Bruker Kappa APEXII area-detector diffractometer
Absorption correction	Multi-scan (*SADABS*; Krause *et al.*, 2015 ▸)
*T*_min_, *T*_max_	0.909, 1.000
No. of measured, independent and observed [*I* > 2σ(*I*)] reflections	25901, 6803, 4957
*R*_int_	0.038
(sin θ/λ)_max_ (Å^−1^)	0.703

Refinement	
*R*[*F*^2^ > 2σ(*F*^2^)], *wR*(*F*^2^), *S*	0.050, 0.130, 1.04
No. of reflections	6803
No. of parameters	334
H-atom treatment	H-atom parameters constrained
Δρ_max_, Δρ_min_ (e Å^−3^)	0.36, −0.27

Computer programs: *APEX4* and *SAINT* (Bruker, 2021 ▸), *SHELXT2018/1* (Sheldrick, 2015*a* ▸) and *SHELXL2018/3* (Sheldrick,2015*b* ▸).

## supplementary crystallographic information

### 3-(14H-Dibenzo[a,j]xanthen-14-yl)phenyl nicotinate Crystal data

**Table:**

C_33_H_21_NO_3_	*Z* = 2
*M**_r_* = 479.51	*F*(000) = 500
Triclinic, *P*1	*D*_x_ = 1.366 Mg m^−^^3^
*a* = 9.6572 (6) Å	Mo *K*α radiation, λ = 0.71073 Å
*b* = 11.0020 (7) Å	Cell parameters from 5269 reflections
*c* = 12.2442 (8) Å	θ = 2.5–30.1°
α = 90.489 (2)°	µ = 0.09 mm^−^^1^
β = 100.530 (2)°	*T* = 100 K
γ = 113.735 (2)°	Bulk, yellow
*V* = 1165.95 (13) Å^3^	0.36 × 0.32 × 0.20 mm

### 3-(14H-Dibenzo[a,j]xanthen-14-yl)phenyl nicotinate Data collection

**Table:**

Bruker Kappa APEXII area-detector diffractometer	4957 reflections with *I* > 2σ(*I*)
φ and ω scans	*R*_int_ = 0.038
Absorption correction: multi-scan (SADABS; Krause *et al.*, 2015)	θ_max_ = 30.0°, θ_min_ = 3.7°
*T*_min_ = 0.909, *T*_max_ = 1.000	*h* = −13→13
25901 measured reflections	*k* = −15→15
6803 independent reflections	*l* = −17→17

### 3-(14H-Dibenzo[a,j]xanthen-14-yl)phenyl nicotinate Refinement

**Table:**

Refinement on *F*^2^	0 restraints
Least-squares matrix: full	Hydrogen site location: inferred from neighbouring sites
*R*[*F*^2^ > 2σ(*F*^2^)] = 0.050	H-atom parameters constrained
*wR*(*F*^2^) = 0.130	*w* = 1/[σ^2^(*F*_o_^2^) + (0.0569*P*)^2^ + 0.312*P*] where *P* = (*F*_o_^2^ + 2*F*_c_^2^)/3
*S* = 1.04	(Δ/σ)_max_ = 0.001
6803 reflections	Δρ_max_ = 0.36 e Å^−^^3^
334 parameters	Δρ_min_ = −0.27 e Å^−^^3^

### 3-(14H-Dibenzo[a,j]xanthen-14-yl)phenyl nicotinate Special details

**Table:**

Geometry. All esds (except the esd in the dihedral angle between two l.s. planes) are estimated using the full covariance matrix. The cell esds are taken into account individually in the estimation of esds in distances, angles and torsion angles; correlations between esds in cell parameters are only used when they are defined by crystal symmetry. An approximate (isotropic) treatment of cell esds is used for estimating esds involving l.s. planes.

### 3-(14H-Dibenzo[a,j]xanthen-14-yl)phenyl nicotinate Fractional atomic coordinates and isotropic or equivalent isotropic displacement parameters (Å^2^)

**Table:**

	*x*	*y*	*z*	*U*_iso_*/*U*_eq_	
O1	0.35504 (11)	0.18818 (10)	0.59813 (8)	0.0178 (2)	
O2	0.22229 (13)	0.30643 (11)	0.52367 (9)	0.0268 (3)	
O7	0.87923 (11)	0.80476 (10)	0.97585 (8)	0.0197 (2)	
N33	−0.03797 (14)	−0.00447 (13)	0.27362 (10)	0.0240 (3)	
C1	0.47627 (15)	0.69861 (14)	0.63629 (11)	0.0178 (3)	
H1	0.507002	0.636540	0.604908	0.021*	
C2	0.35290 (17)	0.71924 (16)	0.57853 (13)	0.0231 (3)	
H2	0.300122	0.672290	0.507435	0.028*	
C3	0.30411 (18)	0.80943 (17)	0.62389 (14)	0.0273 (3)	
H3	0.217241	0.821732	0.584117	0.033*	
C4	0.38106 (18)	0.87912 (16)	0.72467 (13)	0.0264 (3)	
H4	0.347351	0.940087	0.754325	0.032*	
C4A	0.51087 (17)	0.86235 (14)	0.78630 (12)	0.0205 (3)	
C5	0.59429 (18)	0.93596 (15)	0.89061 (12)	0.0228 (3)	
H5	0.564331	0.999844	0.919788	0.027*	
C6	0.71636 (17)	0.91648 (14)	0.94940 (12)	0.0208 (3)	
H6	0.772106	0.967046	1.018663	0.025*	
C6A	0.75951 (15)	0.81993 (14)	0.90623 (11)	0.0166 (3)	
C7A	0.94857 (15)	0.73190 (14)	0.93320 (11)	0.0165 (3)	
C8	1.08433 (16)	0.73893 (15)	1.00455 (12)	0.0211 (3)	
H8	1.119154	0.788960	1.075283	0.025*	
C9	1.16494 (16)	0.67390 (16)	0.97169 (12)	0.0220 (3)	
H9	1.255257	0.677073	1.020378	0.026*	
C9A	1.11560 (15)	0.60092 (14)	0.86498 (11)	0.0176 (3)	
C10	1.20082 (16)	0.53526 (16)	0.82859 (12)	0.0214 (3)	
H10	1.292321	0.539734	0.876516	0.026*	
C11	1.15424 (17)	0.46559 (15)	0.72602 (12)	0.0217 (3)	
H11	1.211989	0.421020	0.703401	0.026*	
C12	1.01996 (16)	0.46040 (14)	0.65411 (12)	0.0197 (3)	
H12	0.988203	0.413047	0.582357	0.024*	
C13	0.93423 (15)	0.52296 (14)	0.68640 (11)	0.0163 (3)	
H13	0.844413	0.518777	0.636413	0.020*	
C13A	0.97798 (14)	0.59385 (13)	0.79350 (11)	0.0148 (3)	
C13B	0.89072 (14)	0.65936 (13)	0.83093 (10)	0.0135 (2)	
C14	0.73410 (14)	0.64189 (13)	0.76384 (10)	0.0133 (2)	
H14	0.741961	0.652772	0.683900	0.016*	
C14A	0.68637 (15)	0.74707 (13)	0.80519 (11)	0.0142 (3)	
C14B	0.55874 (15)	0.76831 (13)	0.74202 (11)	0.0156 (3)	
C15	0.24985 (15)	0.20929 (14)	0.51993 (11)	0.0168 (3)	
C21	0.61320 (14)	0.50198 (13)	0.77181 (10)	0.0125 (2)	
C22	0.53824 (14)	0.40936 (13)	0.67892 (11)	0.0141 (2)	
H22	0.561787	0.431881	0.607783	0.017*	
C23	0.42918 (14)	0.28426 (13)	0.69094 (11)	0.0145 (3)	
C24	0.39272 (15)	0.24641 (14)	0.79284 (11)	0.0163 (3)	
H24	0.318413	0.159754	0.799487	0.020*	
C25	0.46809 (15)	0.33900 (14)	0.88559 (11)	0.0164 (3)	
H25	0.445026	0.315436	0.956668	0.020*	
C26	0.57622 (15)	0.46498 (13)	0.87525 (11)	0.0149 (3)	
H26	0.626011	0.527182	0.939294	0.018*	
C31	0.17384 (15)	0.09795 (14)	0.42980 (11)	0.0166 (3)	
C32	0.04136 (16)	0.09133 (15)	0.35726 (12)	0.0197 (3)	
H32	0.005382	0.158391	0.367709	0.024*	
C34	0.01715 (17)	−0.09640 (16)	0.26070 (12)	0.0248 (3)	
H34	−0.038313	−0.166011	0.202481	0.030*	
C35	0.14949 (18)	−0.09653 (15)	0.32658 (12)	0.0247 (3)	
H35	0.184537	−0.163285	0.312578	0.030*	
C36	0.23056 (17)	0.00303 (14)	0.41390 (12)	0.0203 (3)	
H36	0.321792	0.005858	0.461068	0.024*	

### 3-(14H-Dibenzo[a,j]xanthen-14-yl)phenyl nicotinate Atomic displacement parameters (Å^2^)

**Table:**

	*U*^11^	*U*^22^	*U*^33^	*U*^12^	*U*^13^	*U*^23^
O1	0.0187 (5)	0.0137 (5)	0.0165 (5)	0.0047 (4)	−0.0019 (4)	−0.0035 (4)
O2	0.0337 (6)	0.0250 (6)	0.0225 (5)	0.0176 (5)	−0.0046 (4)	−0.0069 (4)
O7	0.0228 (5)	0.0195 (5)	0.0163 (5)	0.0096 (4)	0.0012 (4)	−0.0056 (4)
N33	0.0182 (6)	0.0237 (7)	0.0209 (6)	0.0005 (5)	0.0014 (5)	−0.0035 (5)
C1	0.0171 (6)	0.0177 (7)	0.0207 (7)	0.0086 (6)	0.0058 (5)	0.0034 (5)
C2	0.0210 (7)	0.0278 (8)	0.0235 (7)	0.0129 (6)	0.0048 (6)	0.0063 (6)
C3	0.0255 (7)	0.0337 (9)	0.0321 (8)	0.0205 (7)	0.0084 (6)	0.0126 (7)
C4	0.0323 (8)	0.0280 (9)	0.0326 (8)	0.0227 (7)	0.0150 (7)	0.0101 (7)
C4A	0.0249 (7)	0.0183 (7)	0.0251 (7)	0.0122 (6)	0.0133 (6)	0.0077 (6)
C5	0.0332 (8)	0.0167 (7)	0.0266 (8)	0.0141 (6)	0.0164 (6)	0.0033 (6)
C6	0.0295 (7)	0.0142 (7)	0.0196 (7)	0.0076 (6)	0.0105 (6)	−0.0002 (5)
C6A	0.0186 (6)	0.0142 (7)	0.0168 (6)	0.0055 (5)	0.0062 (5)	0.0016 (5)
C7A	0.0164 (6)	0.0141 (7)	0.0172 (6)	0.0044 (5)	0.0037 (5)	−0.0003 (5)
C8	0.0194 (7)	0.0229 (8)	0.0159 (6)	0.0054 (6)	−0.0009 (5)	−0.0025 (5)
C9	0.0158 (6)	0.0274 (8)	0.0192 (7)	0.0078 (6)	−0.0025 (5)	0.0001 (6)
C9A	0.0156 (6)	0.0189 (7)	0.0176 (6)	0.0064 (5)	0.0032 (5)	0.0033 (5)
C10	0.0170 (6)	0.0263 (8)	0.0230 (7)	0.0116 (6)	0.0029 (5)	0.0046 (6)
C11	0.0223 (7)	0.0230 (8)	0.0256 (7)	0.0133 (6)	0.0090 (6)	0.0048 (6)
C12	0.0196 (6)	0.0199 (7)	0.0197 (7)	0.0082 (6)	0.0041 (5)	−0.0007 (5)
C13	0.0146 (6)	0.0169 (7)	0.0173 (6)	0.0066 (5)	0.0025 (5)	0.0001 (5)
C13A	0.0130 (6)	0.0131 (7)	0.0161 (6)	0.0033 (5)	0.0026 (5)	0.0018 (5)
C13B	0.0123 (5)	0.0125 (6)	0.0139 (6)	0.0035 (5)	0.0020 (5)	0.0014 (5)
C14	0.0134 (6)	0.0130 (6)	0.0130 (6)	0.0048 (5)	0.0031 (5)	−0.0006 (5)
C14A	0.0154 (6)	0.0108 (6)	0.0169 (6)	0.0043 (5)	0.0071 (5)	0.0026 (5)
C14B	0.0172 (6)	0.0129 (6)	0.0187 (6)	0.0061 (5)	0.0083 (5)	0.0049 (5)
C15	0.0176 (6)	0.0167 (7)	0.0146 (6)	0.0052 (5)	0.0038 (5)	−0.0002 (5)
C21	0.0111 (5)	0.0118 (6)	0.0150 (6)	0.0054 (5)	0.0021 (5)	0.0011 (5)
C22	0.0150 (6)	0.0140 (6)	0.0134 (6)	0.0061 (5)	0.0025 (5)	−0.0001 (5)
C23	0.0141 (6)	0.0126 (6)	0.0154 (6)	0.0055 (5)	−0.0005 (5)	−0.0029 (5)
C24	0.0148 (6)	0.0123 (7)	0.0203 (7)	0.0042 (5)	0.0035 (5)	0.0016 (5)
C25	0.0176 (6)	0.0159 (7)	0.0156 (6)	0.0061 (5)	0.0052 (5)	0.0027 (5)
C26	0.0154 (6)	0.0146 (7)	0.0139 (6)	0.0059 (5)	0.0019 (5)	−0.0010 (5)
C31	0.0166 (6)	0.0148 (7)	0.0139 (6)	0.0012 (5)	0.0043 (5)	−0.0006 (5)
C32	0.0166 (6)	0.0191 (7)	0.0194 (7)	0.0031 (6)	0.0041 (5)	−0.0007 (5)
C34	0.0245 (7)	0.0199 (8)	0.0209 (7)	0.0002 (6)	0.0040 (6)	−0.0060 (6)
C35	0.0294 (8)	0.0176 (8)	0.0237 (7)	0.0068 (6)	0.0044 (6)	−0.0040 (6)
C36	0.0214 (7)	0.0161 (7)	0.0192 (7)	0.0046 (6)	0.0019 (5)	−0.0003 (5)

### 3-(14H-Dibenzo[a,j]xanthen-14-yl)phenyl nicotinate Geometric parameters (Å, º)

**Table:**

O1—C15	1.3567 (16)	C10—H10	0.9500
O1—C23	1.4122 (15)	C11—C12	1.408 (2)
O2—C15	1.2032 (17)	C11—H11	0.9500
O7—C6A	1.3743 (16)	C12—C13	1.3745 (17)
O7—C7A	1.3844 (15)	C12—H12	0.9500
N33—C32	1.3384 (18)	C13—C13A	1.4216 (18)
N33—C34	1.339 (2)	C13—H13	0.9500
C1—C2	1.3725 (19)	C13A—C13B	1.4367 (17)
C1—C14B	1.4187 (19)	C13B—C14	1.5194 (17)
C1—H1	0.9500	C14—C14A	1.5208 (17)
C2—C3	1.406 (2)	C14—C21	1.5295 (18)
C2—H2	0.9500	C14—H14	1.0000
C3—C4	1.361 (2)	C14A—C14B	1.4347 (18)
C3—H3	0.9500	C15—C31	1.4880 (18)
C4—C4A	1.420 (2)	C21—C22	1.3933 (17)
C4—H4	0.9500	C21—C26	1.3981 (17)
C4A—C5	1.420 (2)	C22—C23	1.3851 (19)
C4A—C14B	1.4301 (18)	C22—H22	0.9500
C5—C6	1.360 (2)	C23—C24	1.3805 (18)
C5—H5	0.9500	C24—C25	1.3922 (18)
C6—C6A	1.4172 (18)	C24—H24	0.9500
C6—H6	0.9500	C25—C26	1.3834 (19)
C6A—C14A	1.3711 (18)	C25—H25	0.9500
C7A—C13B	1.3731 (18)	C26—H26	0.9500
C7A—C8	1.4095 (19)	C31—C36	1.3872 (19)
C8—C9	1.356 (2)	C31—C32	1.3926 (19)
C8—H8	0.9500	C32—H32	0.9500
C9—C9A	1.4232 (19)	C34—C35	1.382 (2)
C9—H9	0.9500	C34—H34	0.9500
C9A—C10	1.4159 (18)	C35—C36	1.393 (2)
C9A—C13A	1.4243 (18)	C35—H35	0.9500
C10—C11	1.366 (2)	C36—H36	0.9500
			
C15—O1—C23	116.74 (10)	C9A—C13A—C13B	119.39 (12)
C6A—O7—C7A	117.96 (10)	C7A—C13B—C13A	117.87 (12)
C32—N33—C34	116.56 (13)	C7A—C13B—C14	120.54 (11)
C2—C1—C14B	121.20 (12)	C13A—C13B—C14	121.46 (11)
C2—C1—H1	119.4	C13B—C14—C14A	110.45 (10)
C14B—C1—H1	119.4	C13B—C14—C21	110.01 (10)
C1—C2—C3	120.43 (14)	C14A—C14—C21	110.47 (10)
C1—C2—H2	119.8	C13B—C14—H14	108.6
C3—C2—H2	119.8	C14A—C14—H14	108.6
C4—C3—C2	120.15 (14)	C21—C14—H14	108.6
C4—C3—H3	119.9	C6A—C14A—C14B	118.13 (11)
C2—C3—H3	119.9	C6A—C14A—C14	120.92 (11)
C3—C4—C4A	121.19 (13)	C14B—C14A—C14	120.88 (11)
C3—C4—H4	119.4	C1—C14B—C4A	118.03 (12)
C4A—C4—H4	119.4	C1—C14B—C14A	122.43 (11)
C5—C4A—C4	121.98 (13)	C4A—C14B—C14A	119.54 (12)
C5—C4A—C14B	119.05 (13)	O2—C15—O1	123.75 (12)
C4—C4A—C14B	118.97 (13)	O2—C15—C31	124.61 (13)
C6—C5—C4A	121.07 (12)	O1—C15—C31	111.64 (11)
C6—C5—H5	119.5	C22—C21—C26	118.58 (12)
C4A—C5—H5	119.5	C22—C21—C14	122.07 (11)
C5—C6—C6A	119.29 (13)	C26—C21—C14	119.35 (11)
C5—C6—H6	120.4	C23—C22—C21	119.59 (12)
C6A—C6—H6	120.4	C23—C22—H22	120.2
C14A—C6A—O7	123.36 (11)	C21—C22—H22	120.2
C14A—C6A—C6	122.87 (13)	C24—C23—C22	122.27 (12)
O7—C6A—C6	113.75 (12)	C24—C23—O1	117.43 (12)
C13B—C7A—O7	123.25 (12)	C22—C23—O1	120.26 (11)
C13B—C7A—C8	122.98 (12)	C23—C24—C25	118.03 (13)
O7—C7A—C8	113.77 (11)	C23—C24—H24	121.0
C9—C8—C7A	119.63 (13)	C25—C24—H24	121.0
C9—C8—H8	120.2	C26—C25—C24	120.65 (12)
C7A—C8—H8	120.2	C26—C25—H25	119.7
C8—C9—C9A	120.66 (13)	C24—C25—H25	119.7
C8—C9—H9	119.7	C25—C26—C21	120.87 (12)
C9A—C9—H9	119.7	C25—C26—H26	119.6
C10—C9A—C9	121.21 (12)	C21—C26—H26	119.6
C10—C9A—C13A	119.42 (12)	C36—C31—C32	119.05 (13)
C9—C9A—C13A	119.37 (12)	C36—C31—C15	123.06 (12)
C11—C10—C9A	121.39 (13)	C32—C31—C15	117.88 (12)
C11—C10—H10	119.3	N33—C32—C31	123.51 (13)
C9A—C10—H10	119.3	N33—C32—H32	118.2
C10—C11—C12	119.46 (12)	C31—C32—H32	118.2
C10—C11—H11	120.3	N33—C34—C35	124.22 (13)
C12—C11—H11	120.3	N33—C34—H34	117.9
C13—C12—C11	120.85 (13)	C35—C34—H34	117.9
C13—C12—H12	119.6	C34—C35—C36	118.76 (14)
C11—C12—H12	119.6	C34—C35—H35	120.6
C12—C13—C13A	120.98 (12)	C36—C35—H35	120.6
C12—C13—H13	119.5	C31—C36—C35	117.88 (13)
C13A—C13—H13	119.5	C31—C36—H36	121.1
C13—C13A—C9A	117.87 (11)	C35—C36—H36	121.1
C13—C13A—C13B	122.73 (12)		
			
C14B—C1—C2—C3	−0.8 (2)	C6—C6A—C14A—C14	−178.33 (12)
C1—C2—C3—C4	1.4 (2)	C13B—C14—C14A—C6A	−15.41 (17)
C2—C3—C4—C4A	−0.3 (2)	C21—C14—C14A—C6A	106.54 (14)
C3—C4—C4A—C5	178.83 (14)	C13B—C14—C14A—C14B	167.62 (11)
C3—C4—C4A—C14B	−1.3 (2)	C21—C14—C14A—C14B	−70.44 (15)
C4—C4A—C5—C6	178.65 (14)	C2—C1—C14B—C4A	−0.8 (2)
C14B—C4A—C5—C6	−1.2 (2)	C2—C1—C14B—C14A	178.87 (13)
C4A—C5—C6—C6A	−0.8 (2)	C5—C4A—C14B—C1	−178.31 (12)
C7A—O7—C6A—C14A	12.85 (19)	C4—C4A—C14B—C1	1.8 (2)
C7A—O7—C6A—C6	−168.24 (12)	C5—C4A—C14B—C14A	2.0 (2)
C5—C6—C6A—C14A	2.1 (2)	C4—C4A—C14B—C14A	−177.87 (13)
C5—C6—C6A—O7	−176.83 (12)	C6A—C14A—C14B—C1	179.54 (12)
C6A—O7—C7A—C13B	−9.29 (19)	C14—C14A—C14B—C1	−3.40 (19)
C6A—O7—C7A—C8	170.75 (12)	C6A—C14A—C14B—C4A	−0.78 (19)
C13B—C7A—C8—C9	1.2 (2)	C14—C14A—C14B—C4A	176.28 (12)
O7—C7A—C8—C9	−178.81 (13)	C23—O1—C15—O2	3.13 (19)
C7A—C8—C9—C9A	1.3 (2)	C23—O1—C15—C31	−176.91 (10)
C8—C9—C9A—C10	178.45 (14)	C13B—C14—C21—C22	−115.74 (12)
C8—C9—C9A—C13A	−1.5 (2)	C14A—C14—C21—C22	122.06 (12)
C9—C9A—C10—C11	−179.70 (14)	C13B—C14—C21—C26	64.17 (14)
C13A—C9A—C10—C11	0.3 (2)	C14A—C14—C21—C26	−58.03 (15)
C9A—C10—C11—C12	0.9 (2)	C26—C21—C22—C23	0.39 (18)
C10—C11—C12—C13	−0.9 (2)	C14—C21—C22—C23	−179.70 (11)
C11—C12—C13—C13A	−0.5 (2)	C21—C22—C23—C24	−0.92 (19)
C12—C13—C13A—C9A	1.7 (2)	C21—C22—C23—O1	−178.41 (11)
C12—C13—C13A—C13B	−179.31 (13)	C15—O1—C23—C24	110.45 (13)
C10—C9A—C13A—C13	−1.6 (2)	C15—O1—C23—C22	−71.93 (15)
C9—C9A—C13A—C13	178.43 (13)	C22—C23—C24—C25	0.73 (19)
C10—C9A—C13A—C13B	179.37 (12)	O1—C23—C24—C25	178.29 (11)
C9—C9A—C13A—C13B	−0.6 (2)	C23—C24—C25—C26	−0.03 (19)
O7—C7A—C13B—C13A	176.69 (12)	C24—C25—C26—C21	−0.47 (19)
C8—C7A—C13B—C13A	−3.3 (2)	C22—C21—C26—C25	0.29 (18)
O7—C7A—C13B—C14	−7.4 (2)	C14—C21—C26—C25	−179.62 (11)
C8—C7A—C13B—C14	172.59 (13)	O2—C15—C31—C36	165.16 (14)
C13—C13A—C13B—C7A	−176.04 (13)	O1—C15—C31—C36	−14.80 (18)
C9A—C13A—C13B—C7A	2.98 (19)	O2—C15—C31—C32	−13.6 (2)
C13—C13A—C13B—C14	8.1 (2)	O1—C15—C31—C32	166.49 (11)
C9A—C13A—C13B—C14	−172.91 (12)	C34—N33—C32—C31	−0.7 (2)
C7A—C13B—C14—C14A	18.63 (17)	C36—C31—C32—N33	1.8 (2)
C13A—C13B—C14—C14A	−165.59 (11)	C15—C31—C32—N33	−179.43 (13)
C7A—C13B—C14—C21	−103.59 (14)	C32—N33—C34—C35	−0.9 (2)
C13A—C13B—C14—C21	72.20 (14)	N33—C34—C35—C36	1.4 (2)
O7—C6A—C14A—C14B	177.54 (12)	C32—C31—C36—C35	−1.2 (2)
C6—C6A—C14A—C14B	−1.3 (2)	C15—C31—C36—C35	−179.93 (13)
O7—C6A—C14A—C14	0.5 (2)	C34—C35—C36—C31	−0.2 (2)

### 3-(14H-Dibenzo[a,j]xanthen-14-yl)phenyl nicotinate Hydrogen-bond geometry (Å, º)

*Cg*4 and *C*g7 are the centroids of the C4*A*/C5/C6/C6*A*/C14*A*/C14b*B* and C21–C26 rings, respectively.

**Table:**

*D*—H···*A*	*D*—H	H···*A*	*D*···*A*	*D*—H···*A*
C24—H24···N33^i^	0.95	2.53	3.3298 (18)	142
C11—H11···O2^ii^	0.95	2.56	3.329 (2)	138
C11—H11···*Cg*7^iii^	0.95	3.05	3.8352 (19)	142
C25—H25···*Cg*4^iv^	0.95	3.01	3.9301 (15)	162

Symmetry codes: (i) −*x*, −*y*, −*z*+1; (ii) *x*+1, *y*, *z*; (iii) *x*−1, *y*, *z*; (iv) −*x*+1, −*y*+1, −*z*.
