# Supplementary material for: Synthesis and structure of 3-(14H-dibenzo[a,j]xanthen-14-yl)phenyl nicotinate
Source: Acta Crystallogr E Crystallogr Commun. 2026 Apr 10;82(Pt 5):437–40. doi: 10.1107/S2056989026003415 (PMC13148218; doi:10.1107/S2056989026003415)
Supplement: Supplementary file 3 [file e-82-00437-sup4.docx]

**Spectroscopic data (IR and NMR):**

**IR** (KBr), \v (cm^-1^): 3070, 2919, 2852, 1728 (C\\dbO~ester~), 1637, 1622, 1607, 1589, 1514, 1486, 1459, 1430, 1401, 1272, 1252, 1230, 1218, 1134, 1090, 1075, 1021, 965, 821, 800, 733, 698. **^1^H NMR** (500 MHz, DMSO-d6, 301 K) (J, HZ): \d 6.53 (s, 1H, 14-CH), 6.91 (ddd, 1H, H phenol, J = 8.1, 2.3, 0.8), 7.23 (t, 1H, H phenol, J = 8.0), 7.34--7.36 (m, 1H, H phenol, J = 8.0), 7.36--7.39 (m, 1H, H phenol), 7.43 (t, 2H, H Ar, J = 7.5, 7.0, 0.7), 7.49 (d, 2H, H Ar, J = 9.0),

7.48-7.53 (m, 1H, H Py), 7.61 (t, 2H, H Ar, J = 7.7, 7.0, 1.2), 7.80 (d, 2H, H Ar, J = 8.9), 7.83 (d, 2H, H Ar, J = 7.8), 8.31 (dt, 1H, H Py, J = 8.0, 2.0), 8.38 (d, 2H, H Ar, J = 8.5), 8.8 (dd, 1H, H Py, J = 4.8, 1.7), 9.3 (d, 1H, H Py, J = 1.9) ppm. **^13^C NMR** (125 MHz, DMSO-d6, 301 K) (J, Hz): \d 37.92 (14-CH), 116.88 (2C), 118.15 (2CH Ar), 119.81 (1CH phenol), 121.24 (1CH phenol), 122.64 (2CH Ar), 123.49 (1CH phenol), 124.53 (2CH Ar), 125.68 (1C), 126.08 (1CH Py), 127.11 (2CH Ar), 129.00 (2CH Ar), 129.25 (2CH Ar), 129.50 (1CH phenol), 131.17 (2C), 131.45 (2C), 137.68 (1CH Py), 146.90 (1C) 148.90 (2C), 150.73 (1C), 151.49 (1CH Py), 154.01 (1CH Py), 163.55 (C\\dbO) ppm. Found: C, 82.85; H, 4.42; N, 2.90. C~33~H~21~NO~3~ requires C, 82.66, h, 4.41; N, 2.92 %.
